# A Pilot Study to Examine If Dietary Habits Can Affect Symptomology in Mild Pre-Vaccination COVID-19 Cases

**DOI:** 10.3390/biology11091274

**Published:** 2022-08-27

**Authors:** Kaine Moreno McDaid, Mridula Chopra

**Affiliations:** 1 Institute of Biomedical and Biomolecular Sciences (IBBS), School of Pharmacy and Biomedical Sciences, University of Portsmouth, Portsmouth PO1 2DT, UK; 2 Fundación Euroinnova, Edificio INNOVA, Camino de la Torrecilla 30, 18200 Maracena, Spain

**Keywords:** COVID-19, coronavirus, SARS-CoV-2, diet, food frequency questionnaire, IgG, symptoms, long-COVID

## Abstract

**Simple Summary:**

COVID-19 related symptoms and severity of infection vary greatly among individuals and althoμgh various factors including age, gender, obesity, and underlying co-morbidities are sμggested to be the confounding variables that affect the severity of disease, a small percentage of apparently healthy and young people also succumb to disease, this was especially true before the COVID-19 vaccines were developed. Recently, few studies have sμggested that COVID-19 severity is lower in individuals who followed a plant based and pescatarian diet. Aim of the current study was to examine whether symptoms experienced by COVID-19 patients can be predictive of mild and long-term disease pre-vaccination and if the intake of specific nutrients as well as serum 25(OH)D concentrations are related to the severity of symptoms experienced by individuals. Additionally, we performed longitudinal and cross-sectional analysis on antibodies against SARS-CoV-2 to examine the duration and degree of protection conferred by natural immunity after SARS-CoV-2 infection. We found several nutrients such as vitamin E, poly-unsaturated fatty acids, and fibre amongst others to be possibly linked to a reduced COVID-19 severity.

**Abstract:**

The heterogeneity of the severity of symptoms of COVID-19 experienced by the young and healthy individuals is poorly understood. The present study was undertaken to mainly examine whether the respective diets and the type of symptoms experienced by patients are predictive of having long COVID-19. Disease severity was assessed with a symptomatology questionnaire and used to group 55 participants in asymptomatic (AS), mild symptoms (S) and long COVID (LC). We found that experiencing a higher number of symptoms as well as fatigue were predictors of developing LC whereas those who experienced rhinorrhea were less likely to develop LC. Blood samples were also taken to measure vitamin D [25(OH)D] concentrations and duration of spike IgG antibodies. In this study, serum 25(OH)D was not significantly different between 3 symptom groups with median (IQR) ng/mL levels of 22.0 (12.3) in the AS, 22.3 (7.5) in S, and 24.9 (9.4) in the LC group (*p* ≥ 0.05). The duration of IgG antibody response was found to vary greatly, with some individuals showing raised IgG over a year after infection. To examine whether dietary factors can influence the severity of symptoms, diet was analysed using 4–7-day food diaries as well as a Food Frequency Questionnaire (FFQ). Some nutrients such as vitamin E, polyunsaturated fatty acids, fibre, and iron were associated with lower severity of COVID-19. Lower intake of vitamin E was associated with having LC with a median (IQR) intake of 6.2 mg (3.8) seen in LC vs. 8.6 mg (7.2) in the AS group (*p* = 0.047). This pilot study has highlighted a few differences in the number and type of symptoms experienced by the young non-hospitalised individuals with mild and long COVID-19 and identified a few dietary components for their potential protective role against long COVID-19, however, the findings need to be confirmed with further large scale studies.

## 1. Introduction

In late December of 2019, the first cases of a new respiratory infection of unknown etiology were identified in Wuhan, China. This virus was later named SARS-CoV-2 (severe acute respiratory syndrome coronavirus 2) and the disease caused by the virus as coronavirus disease 2019 (COVID-19). SARS-CoV-2 has reportedly claimed the lives of more than 6 million people to date [1]. Vaccine rollout has been impressively fast and now over 4.7 billion people have been fully vaccinated, however, it is still necessary to continue examining the causes of the heterogeneity of severity of symptoms seen with COVID-19 infection, as this may help us prepare better for any future disease outbreaks, COVID-19 related or otherwise.

Mild illness with COVID-19 lasts for approximately two weeks, however, some individuals continue to experience symptoms even after they have tested negative for the SARS-CoV-2 virus. According to the definition provided by WHO, post-COVID symptoms (PCS) that persist or return three months after a person is infected with SARS-CoV-2 is defined as “long-term COVID”. Some of the common symptoms experienced by these individuals include chronic headaches, fatigue, myalgia, ‘brain fog’, and persistent loss of taste and smell. These individuals can often be young and healthy and are not considered ‘at risk’ groups for severe COVID-19 [2,3]. The apparent cause of the presence of persistent symptoms in these individuals is not fully known, thoμgh some clues are emerging. For example, individuals with long-term COVID may show different immunological profiles after COVID-19 infection than those who do not suffer long-term COVID. One study showed that subsets of unactivated naive CD4 and CD8 T cells as well as some subsets of B cells were missing in the peripheral blood of individuals with PCS compared to gender and age-matched individuals who did not suffer PCS [4]. A recent paper has sμggested that long-term-haulers demonstrate dysfunctions in T cell memory generation and effector molecules [5]. There are disparities in the reported persistence of antibody seropositivity post-SARS-CoV-2 infection. A study with over 1000 participants indicated that following infection, 91% of individuals show a sustained IgG antibody response for up to 4 months [6]. Similar results were seen in the Mount Sinai hospital where 95% of the participants with mild-moderate symptoms had raised IgG concentrations over 5 months [7]. However, some studies have reported a sharp decline in antibodies just 2 months after a mild infection [8], while others have shown the presence of neutralising antibodies up to 1 year after infection [9]. The reasons behind the disparities in the severity of symptoms and waning of antibodies remain unclear to date, thoμgh level of exposure to the virus, genetic factors, lifestyle and dietary habits of individuals may play a role. 

Few recent studies have sμggested that COVID-19 severity is lower in individuals consuming a plant-based diet [10,11]. Kim et al. [12] examined the diets of healthcare workers across 6 European countries and concluded that plant-based or pescatarian diets that contained high amounts of vegetables, legumes, and nuts as well as lower amounts of poultry and red and processed meats had 59–73% lower odds of developing moderate to severe COVID-19. Several dietary components can regulate inflammation and immune function. A high salt diet has been shown to increase the ACE1/ACE2 ratio promoting inflammation and raising blood pressure [13]. The downstream effects of this can lead to a sustained inflammatory state [14]. Dietary antioxidants such as polyphenols and carotenoids decrease inflammation and oxidative stress. It is worth noting that an efficient function of the immune system relies on the adequate intake of micronutrients. Vitamins B2, B6, B9. B12, A, C, D, and E as well as selenium, iron, copper, and zinc have all been shown to support immune function via mechanisms such as strengthening epithelial barriers and regulating cell-mediated immune responses as well as humoral responses [15]. Vitamin D is known for its immunomodulatory effects. It decreases the production of pro-inflammatory Th17 and promotes the induction of modulatory T regulatory cells [16,17]. This leads to downstream effects causing a decrease in proinflammatory cytokines such as IL-17 and IL-21 and increased concentrations of anti-inflammatory cytokines such as IL10. Vitamin D also plays a role in the protection of the epithelial barrier of the alveoli against acute lung injury [18]. It is therefore feasible that those who are vitamin D deficient may be more susceptible to COVID-19 and therefore can suffer more severe effects of COVID-19 illness as well as potential long-term consequences. In older patients, baseline vitamin D levels have been reported to correlate with the severity of symptoms [19]. A previous meta-analysis demonstrated a protective effect of vitamin D supplementation against respiratory tract infections [20]. In hospitalised COVID-19 patients, supplementation of vitamin D has been reported to improve the clinical outcome in the relatively older age group patients [21,22]. More evidence should be collected to examine whether vitamin D does play a role in COVID-19 severity and whether results obtained from the studies in older populations are also generalizable to younger populations.

In order to better understand factors that may lead to the diversity of symptoms and severity of COVID-19 in non-at-risk adults, we evaluated specific components of diet and immunity using a mixed method study design. The aim of our study was to explore the uncertainties surrounding COVID-19, by looking at the diet, lifestyle, immunity, and vitamin D in non-hospitalised relatively young asymptomatic, symptomatic, and individuals with long-term COVID-19. We have examined whether we can identify specific nutrients that could be associated with the heterogeneity of symptoms and longevity of IgG levels seen in COVID-19 patients.

## 2. Materials and Methods

### 2.1. Study Design

This study has 3 arms (aims A, B and C).

The first one (Aim A) is an observational study, exploring the differences in symptomology of those who present with long-term COVID-19 (LC) to those without LC, and, examine the effect of dietary habits on the severity of COVID-19 in individuals.

The second (Aim B) is a retrospective case–control study analysing the relationship between vitamin D [25(OH)D] concentrations in serum and COVID-19 disease severity.

Finally, third arm (Aim C) involves the cross-sectional and longitudinal analysis of serum IgG antibody concentrations in post-COVID-19 infection and its correlation with dietary components.

The same cohort was involved in all three arms of this study.

Power calculations were computed for this study except for aim A which is a pilot study; therefore, no power was computed. For aim B (retrospective), a level of <20 ng/mL of vitamin D was considered deficiency of this vitamin and is based on the study conducted by Panagiotou et al., where serum vitamin D levels in COVID-19 infected patients were measured [23]. By using the means of intensive care unit (ICU) patient’s vs. non-ICU patients, and with an alpha of 0.05 and a 1-beta of 0.8, it was necessary to recruit minimum of 42 participants for a significant difference to be detected. For aim C, based on the previous research, COVID-19 patients can be differentiated from COVID-19 negative patients based on a serum IgG spike protein (S1) antibody level for COVID-19 of >10 RU/mL which equates to 32 BAU/mL (we used 33.8 BAU/mL as a threshold for positivity as this was recommended by the manufacturer) [24]. Using this criterion, and with an alpha of 0.05 and a 1-beta of 0.8, recruitment of a minimum of 18 participants was necessary to detect a significant difference between those who had or had not seroconverted.

### 2.2. Ethical Considerations

This research was approved by the Junta de Andalucía ethics committee.

#### Participants and Recruitment

All participants provided an informed consent for the study. A total of 56 participants were recruited after considering exclusion and inclusion criteria described below however data from only 55 participants was used for the analysis due to a drop out of one participant.

Inclusion criteria were ages 18–50 years and those who had given a positive result on a COVID-19 test (PCR, antigen, or antibody). This age group was chosen as they are most likely to have subclinical infection.

Exclusion criteria were persons who received a COVID-19 vaccination before testing positive for COVID-19 (13 were excluded as they had received SARS-CoV-2 vaccination before testing positive for the infection). Any persons that required hospitalization due to their COVID-19 infection (1 individual was excluded on this basis) and those with chronic illnesses (3 excluded due to this criteria) that are known to impact the severity of COVID-19 (such as diabetes, cardiovascular disease, cancer, and chronic respiratory disease, i.e., asthma, COPD, etc.) were excluded from the study [25].

Participants were recruited in Granada throμgh the company Euroinnova™ between December 2020 and July 2021 as during this period the company offered employees COVID-19 screening using ELISA and chemiluminescence assays. All employees were offered the possibility to participate but only those who reported a positive result on a COVID-19 test (lateral flow, PCR, antigen, or ELISA) and consented to participation were included in the analysis.

### 2.3. Questionnaires

Participants were asked to complete 2 questionnaires and a food diary:

1. A symptomology questionnaire, used to assess the severity of symptoms, this was a questionnaire that was adapted from a sero-epidemiological questionnaire published by the World Health Organisation [26]. (Table A1)
2. The food frequency questionnaire (FFQ) was used in conjunction with the food diaries to examine the dietary habits of individuals. FFQ estimated intake of different food items prior to COVID-19 infection. Participants were asked to score how many portions they consumed over the course of a month, week, or day, before having COVID-19 (Table A2). This information was then used to calculate portions of food groups consumed by participants per week. The FFQ used has been validated previously in young and healthy Spanish participants [27].
3. Food diaries allowed for the calculation of average consumption of macro/micronutrients per day, and the analysis was done using the Nutritics™ software. The food diaries asked the portions and ingredients of the meals of participants over the course of 4–7 days, with one day being over a weekend or holiday.

All questionnaires were collected via interview or via the app EUResearch™ which was developed to monitor positive COVID-19 cases as well as to collect data for this study.

### 2.4. Blood Sampling

Blood samples were drawn in blood tubes with no anticoagulant and serum samples were analysed during a 6-month period of the study. Each participant was requested to provide a blood sample and where possible analysed twice over the 6-month period, the first samples were taken between December 2020 and April 2021 and the last samples were taken in July 2021. Person analyzing blood samples were blind as to the symptom group of the participants as well as their personal details that can identify them.

SARS-CoV-2 antibodies were detected using a qualitative sandwich immunoassay (ADVIA Centaur COV2T™) which detected if IgM and/or IgG antibodies were present. A quantitative chemiluminescent microparticle immunoassay (CMIA) (ARCHITECT™) assay was used to quantify concentrations of IgG in serum. Briefly, this assay detects IgG antibodies, including neutralising antibodies, to the receptor binding domain (RBD) of the S1 subunit of the spike protein of SARS-CoV-2 in serum. The S1 RBD is responsible for binding to the ACE-2 receptor of the host cell; Therefore, antibodies that target S1 are expected to have neutralisation activity [28]. Vitamin D concentrations were measured using Alinity™ reagent kit. This is also a CMIA used for measuring concentrations of 25(OH)D in serum or plasma.

### 2.5. Study Cohort

Participants were divided into groups to analyse respective differences between COVID-19 severity. Group 1 were asymptomatic (AS), group 2 were symptomatic with no persistent symptoms (S) and group 3 reported being sufferers of long-term COVID (LC). The symptom groups are shown in Table 1.

Table 2 provides demographic and grouping details of the cohort. Out of 55 participants who completed the symptomology questionnaire, 15 were asymptomatic, 15 in the mild symptom category and 25 had long-term COVID. A total of 53 participants gave blood for analysis over the 6-month period. Out of 53 participants, 39 provided their blood samples twice and 13 provided blood samples only once.

### 2.6. Statistical Analyses

The data was analysed using IBM SPSS statistics 26 software. Normal distribution was assessed using Shapiro–Wilk significance where ≤0.05 and skewness value > 0.5 were considered deviations from a normal distribution. Mann–Whitney U tests and Spearman correlations were as the data was skewed, where possible the data was transformed using log_10_ which allowed for parametric tests to be used. Data are presented as mean (SD) where parametric tests were used and median (range), IQR where non-parametric tests were used. Chi squares tests were also performed (Table 3).

Logistic regression analysis was performed using the forwards entry method and was used to predict which individuals developed PCS (Table 4). Logistic regression was also used to predict if a participant suffered a particular symptom, using food groups and nutrients as predictors.

## 3. Results

### 3.1. Symptomology

**Figure 1:**
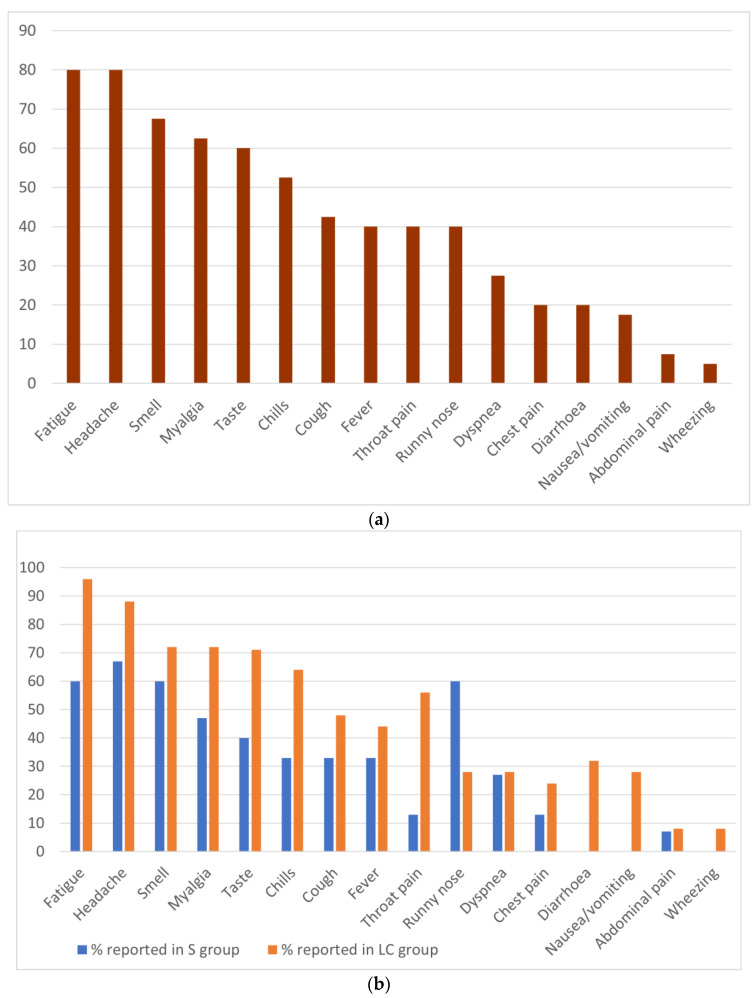
(**a**). Prevalence of symptoms in the cohort; (**b**) Prevalence of symptoms in the mild (S group) and long-term COVID (LC group). (**b**) shows the percentage of participants that experienced each symptom by the mild symptoms (Group S, blue bar) and those who reported having long-term COVID (orange bar).

The most frequently experienced symptoms overall by the individuals with symptoms were fatigue (80.0%), headache (80.0%), loss of sense of smell (67.5%), myalgia (62.5%), and loss of sense of taste taste (60.0%) (Figure 1a). Figure 1b shows a comparison of the prevalence of symptoms experienced by participants from the mild (Group S) and long-term COVID (LC group).

**Figure 2:**
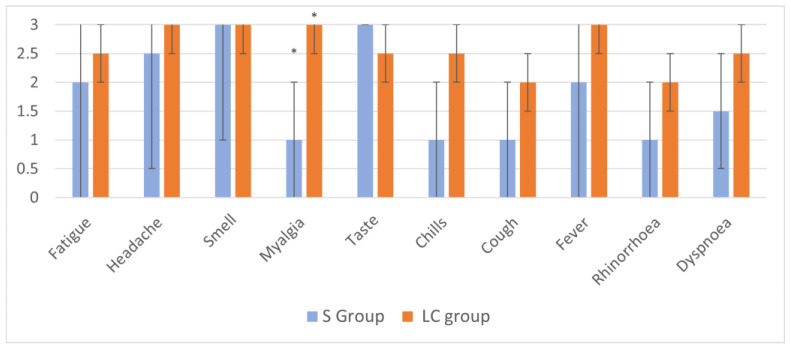
Severity of symptoms experienced by the mild symptoms (S group) and long-term COVID (LC group). The difference in reported severity (0–3) across symptom group. Results shown are me-dian severity of symptoms across symptomatic (S) and long-term COVID groups (LC). Mann–Whitney U tests were performed for each symptom across groups * *p* ≤ 0.05.

Participants were asked to score each individual symptom on a scale from 1 to 3 indicating mild, moderate, and severe, participants were also asked to rate their overall severity of COVID on a scale of 1–10 (1 = least severe and 10 = most severe). Results shown in Figure 2 show that except for the symptoms loss of sense of smell and taste, patients with long-term COVID-19 show higher severity of symptoms and myalgia was significantly more severe between the two groups who experienced symptoms (*p* ≤ 0.05, Mann–Whitney test).

Figure 2 shows differences in the reported severity of each symptom in mild symptomatic and long-term COVID group. It is worth mentioning that the Mann–Whitney U produced significance levels of *p* = 0.050 for fever, 0.052 for chills and 0.072 for dyspnoea. The *p* value for myalgia was 0.019.

Participants were also asked to give an overall severity score of symptoms between 1 and 10. The median (IQR) score for the S group was 3 (4) and for the LC group it was 6 (3), Mann–Whitney U test revealed that LC group experienced significantly higher overall severity of symptoms with COVID-19 infection compared to the S group (*p* = 0.04). The Mann–Whitney U test was then repeated to see if the number of reported symptoms was different between groups. The median (IQR) number of symptoms for the S group was 5 (4) and for the LC group was 7 (4), *p* < 0.01 The total number of symptoms experienced positively correlated with the total severity of symptoms (r = 0.708, *p* < 0.001 spearman correlation).

Chi squared tests were carried out to see if there was a significant difference in gender between the 3 symptom groups, but no significance was seen. Chi square analysis were then run to see if there were significant differences in the type of symptoms reported in the S and LC groups. There was a significant association between the frequency of several symptoms (fatigue, throat pain, nausea/vomiting, and diarrhoea) and occurrence of long-term COVID-19 in participants. Rhinorrhoea was significantly associated with not having long-term COVID in participants (Table 3).

The data in Table 3 sμggests that fatigue and throat pain showed a strong association (Bayes factors > 10) with the presence of long-term COVID in symptomatic individuals as the odds ratios showed that the odds of long-term COVID patients suffering from fatigue and throat pain were 16.75 and 8.23 times higher than mild symptom group respectively. Diarrhoea and nausea/vomiting were moderately associated (Bayes factors between 3 and 10) with the presence of long-term COVID. The presence of a runny nose was weakly associated (Bayes factor < 3) with being part of the mild symptom group without long-term COVID.

Logistic regression was then used to see if the number and type of symptoms experienced could predict which symptoms were significantly associated with the presence of long-term COVID. The symptoms that were significant in the chi square analysis were used in the model with the exceptions of nausea/vomiting and diarrhoea due to 0 frequency counts in the S group. Age, BMI, and gender were also added to the regression model. The model that was significant is shown in Table 4.

Throat pain, age, gender, and BMI were not significant predictors and therefore removed from the model. Fatigue and number of symptoms were a significant predictor of developing long-term COVID. Thoμgh the effect size seen for rhinorrhoea was small, having a runny nose was a significant predictor of not developing long-term COVID. However, it is worth noting that there are big confidence intervals associated with the logisitic regression coefficient and odds ratios for fatigue and rhinorrhoea.

### 3.2. Diet and Symptomology

Results from the FFQ were compared across the 3 symptom groups, and the Mann–Whitney U test was used to look for the significant differences between groups. There were significant differences observed between the consumption of 5 food groups, these are shown in Table 5. Consumption of the food groups that were not significant are shown in Table A3.

We also compared the three groups using data from the food diaries. Table 6 shows differences in consumption of macro and micronutrients across the three symptom groups. Significant differences were seen in the consumption of omega 3, vitamin E and vitamin B12. It should be noted that monounsaturated fat nearly achieved statistical difference between the AS and LC groups (*p* = 0.057).

### 3.3. Food Groups and Nutrients and Symptom Severity

**Figure 3:**
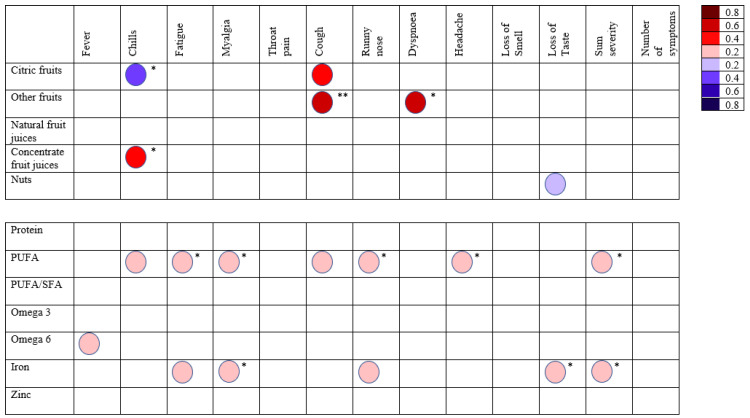

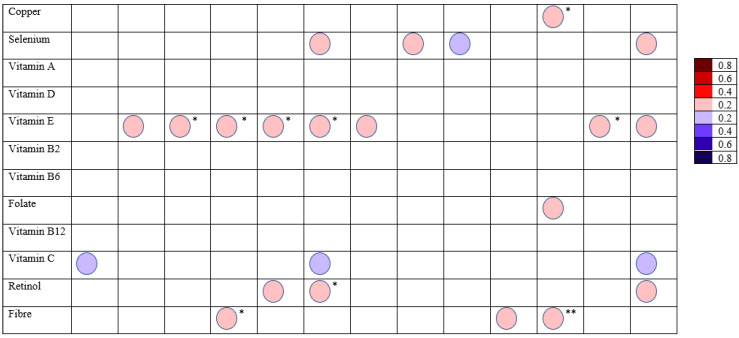
Correlation matrix showing correlations between components of the diet and the severity of individual symptoms as well as the sum of severity scores given by participants and number of symptoms reported by a participant. PUFA—Poly-Unsaturated Fatty Acids; SFA—Saturated Fatty Acids, red shows negative correlations and blue positive correlations. * *p* ≤ 0.05, ** *p* ≤ 0.01, those shown without an asterisk are *p* value of >0.05 but <0.09. Colour intensity indicates the degree or strength of correlation coefficient r, as shown on the right.

We correlated the severity scores for each symptom to chosen food groups from the FFQ and nutrients from the food diaries (Figure 3). Selection of food groups and nutrients was based on those which were thoμght to most likely impact COVID-19 severity. Symptoms were only included if at least ten participants experienced that symptom. The symptoms that were selected were fever (n = 16), chills (n = 21), fatigue (n = 32), myalgia (n = 25), throat pain (n = 16), coμgh (n = 17), rhinorrhoea (n = 16), dyspnoea (n = 10), headache (n = 32), loss of sense of smell (n = 26) and loss of sense of taste (n = 23), the total number of participants who experienced symptoms was 40. We also summed the severity scores to give an overall severity score and we also correlated the number of symptoms with nutrient consumption (Figure 3). In general, many significant negative correlations were seen between symptom severity and intake of poly-unsaturated fatty acids, iron, and vitamin E. Negative correlations of some symptoms were also seen with “other fruits”, “concentrate fruit juices”, copper, retinol, and fibre (Figure 3). A significant positive correlation was found between citric fruit consumption and chills severity (r = 0.490, *p* = 0.024).

We investigated if there were differences in food groups and nutrients across individuals who reported ‘yes’ and ‘no’ to suffering a particular symptom, the results are shown in Table 7 and Table 8. We also investigated if those who presented with symptoms and those who did not, had significant differences in the diet, but no significant difference was seen.

### 3.4. Vitamin D and COVID-19 Severity

Serum levels of 25(OH)D were compared between three groups and results are shown in Table 9. Mann–Whitney U test was performed as the data was not normally distributed. No significant difference was seen for the serum 25(OH)D concentrations across the 3 symptom groups.

### 3.5. Crossectional IgG SARS-CoV-2 Antibody Analysis

Blood was drawn and measured for SARS-CoV-2 IgG antibodies from participants over a 6-month period. During the first sample collection, spike protein IgG antibodies were detectable in 26 individuals but 6 of them were below the threshold for positivity (33.8 BAU/mL). There were another two individuals who had previously tested positive in a PCR test but whose IgG antibodies were completely undetected by the ELISA. During the second sample collection, antibodies were detectable in 29 individuals, but 5 individuals had spike IgG antibody concentrations below the threshold for positivity, another 3 individuals who had tested positive in a PCR test had undetectable IgG concentrations during the ELISA.

**Figure 4:**
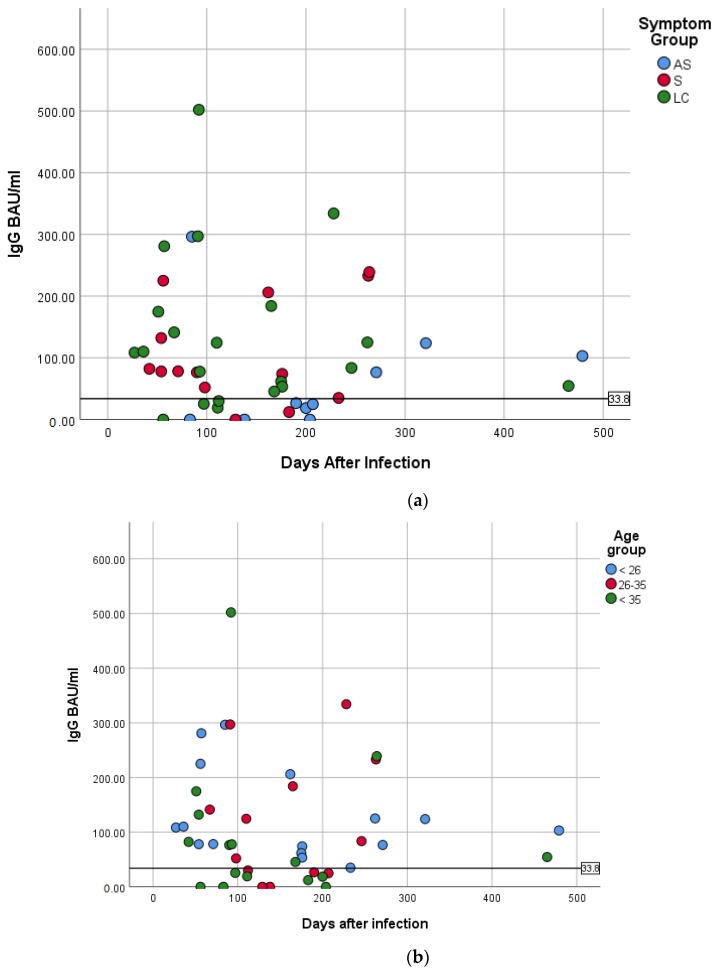
(**a**) Serum IgG concentration over time after infection in 3 COVID-19 groups. Number of days IgG was detectable in three patients’ groups. The reference line on the y axis shows the threshold for positivity (33.8 BAU/mL). (**b**) Serum IgG concentrations over time after infection for different age groups. Longevity of serum IgG antibody is shown in days for different age groups. The reference line on the y axis shows the threshold for positivity (33.8 BAU/mL).

Figure 4a,b show the differences in spike IgG antibodies over time across symptom groups and age groups respectively. Figure 4a shows that the asymptomatic individuals generally had lower antibody concentrations, with many falling below the threshold of positivity.

Figure 4b shows that none of the younger age group (<26) were below the threshold for positivity. We decided to investigate these results further and grouped all participant’s serum IgG results collected over the 6 months period. Mann–Whitney U tests were performed to see if significant differences were observed in the age and symptom groups. Results are shown in AppendixTable A4a,b. The results show that the younger group (≤25) had significantly higher concentrations of spike IgG antibodies than the ≥36 group (*p* = 0.014) and the 26–35 group (*p* = 0.050). When a comparison was made between different symptom groups, the long-term COVID group had significantly higher antibody levels compared to asymptomatic group (*p* = 0.024).

### 3.6. Longitudinal Antibody SARS-CoV-2 IgG Analysis

**Figure 5:**
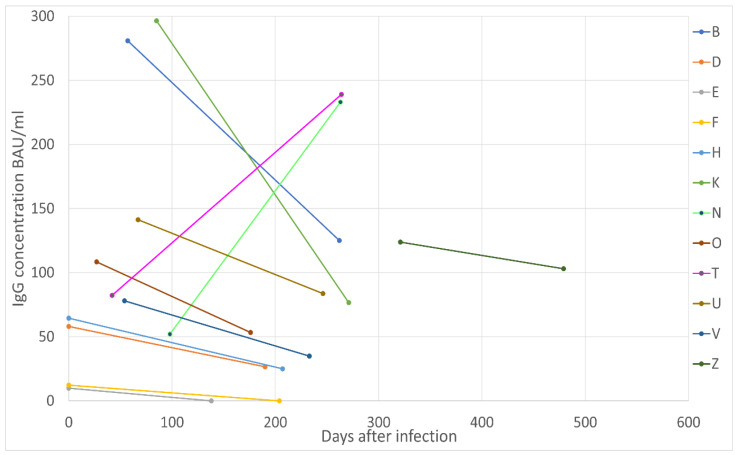
Paired antibody analysis showing serum antibody longevity in days after SARS-CoV-2 infection. Each coloured line represents one of the 12 participants who gave blood twice between December 2020 and July 2021.

From the participants recruited between December 2020 and April 2021, twelve were eligible for the longitudinal antibody analysis. Figure 5 shows a line graph of the paired antibody analysis from 12 participants who had antibodies measured twice over 6 months period. Note that none of the 12 participants received any dose of a COVID-19 vaccine during this time.

Most individuals who participated in the longitudinal antibody analysis showed a steady decline in IgG concentration over time. Only two participants (labelled N and T in Figure 5) showed an increase in IgG concentration. Neither of these participants had received any dose of COVID-19 vaccine at the time of blood sampling. Both E and F had IgG concentrations below the threshold for positivity during the first blood sampling (33.8 BAU/mL) and had undetectable concentrations of spike IgG antibodies during the second sampling.

From the longitudinal analysis we found the minimum duration of IgG antibodies to be 138 days, and the maximum to be 479 (W). Participants D, E, F, K and Z were all asymptomatic. B, O and U were in the LC group while N, T and V were in the S group. Characteristics and dietary intake of different nutrient intake of the 12 participants was obtained from their food diaries and the results are shown in Table A5.

### 3.7. IgG Correlations with Dietary Components

Food groups from the food frequency questionnaire were then correlated to antibody concentrations using a spearman correlation. Correlation coefficients and significance values are shown in Table 10. The food group ‘other fruits: apple, pear, peach, banana’. positively correlated to spike IgG concentration in the whole group. In the asymptomatic group, natural fruit juice consumption negatively correlated to antibody concentration.

Table 10 shows correlation coefficients and significance values for semi partial correlations of weekly portions of citric fruits, other fruits, and natural fruit juices with antibody concentrations, controlling for age and time after infection.

## 4. Discussion

### 4.1. Symptomology

In agreement with previous research, we found that the number of symptoms experienced can be an important predictor of long-term COVID [30]. Our investigation shows that in terms of symptom prevalence (Figure 1b): fatigue, headache, myalgia as well as loss of smell and taste were the most prominent symptoms in the long-term COVID (LC group), which concurs with previous studies [30]. When participants were asked to rank the severity of their symptoms from mild, moderate, and severe, myalgia was found to be significantly more severe in the LC individuals than S individuals. There are multiple potential causes of myalgia in COVID-19 patients. ACE 2 is expressed on skeletal muscle, which means SARS-CoV-2 may bind directly and infect these cells [31]. Alternatively, myalgia may be caused by an uncontrolled inflammatory response known as a cytokine storm. Sustained elevated concentrations of pro-inflammatory cytokines can induce the formation of prostaglandin E2, a pain mediator which affects pain receptors in the peripheral nervous system [31]. Previous studies have reported increased concentrations of IL-6,10 and TNF-α in severe cases of COVID-19 [32,33]. Alternatively, mitochondrial dysfunction can also be a cause of myalgia in individuals [34]. Mitochondrial dysfunction can be triggered by viral infection [34]. which can cause impaired oxidative phosphorylation leading to myalgia, chronic fatigue, and neurocognitive symptoms, all of which can be observed in COVID-19 long-haulers.

Chi square analysis revealed that fatigue, throat pain, nausea/vomiting and diarrhoea were associated with LC group and rhinorrhoea with S group, this was then used to make a logistic regression model, which sμggested fatigue and number of symptoms experienced to be predictors of developing LC whilst rhinorrhoea as a predictor of not developing long-term COVID (Table 3). In agreement with previous studies, fatigue was found to be one of the prominent long-term effects of COVID infection in long-haul patients (Figure 1b) [30]. This was also seen in the SARS 2003 outbreak, from which, many patients reported chronic fatigue up to 12 months following infection [35]. The mechanisms behind chronic fatigue are still not fully understood, it has been sμggested that miscommunication of inflammatory response pathways could play a role, however evidence for this is currently dubious and more research is needed [36].

Results from our study sμggest that rhinorrhoea may be a predictor of not developing LC. Rhinorrhoea has been reported to have a prevalence as high as 60% in COVID-19 positive cases [37,38]. One study examined the replication kinetics of SARS-CoV-2 of human respiratory epithelium both in the presence and absence of co-infections with rhinovirus and showed that SARS-CoV-2 replication was impaired in the presence of rhinovirus infection due to the upregulation of interferons [39]. However, both robust and lack of interferon responses have been reported in severe cases of COVID-19 [40,41]. These studies however measured interferons in the peripheral blood and not in human respiratory epithelium, where their localised effect seems to impair SARS-CoV-2 replication in human respiratory epithelium. It is then feasible that rhinorrhoea could be an indicator of coinfection of SARS-CoV-2 and rhinovirus and individuals with this co-infection are less likely to develop long-term COVID due to an increased localised interferon response in the early stages of infection, however the negative association between rhinorrhoea and long-term COVID needs further investigation. To our knowledge, we are the first to report that the presence of rhinorrhoea may affect the development of long term COVID-19.

Diarrhoea and nausea/vomiting were associated with the presence of long-term COVID in symptomatic individuals. They were not significant contributors to the regression model (Table 4), however previous research has found GI problems such as diarrhoea and abdominal pain to be predictive factors of long-term COVID in individuals [30]. These symptoms may be indicative of multi organ system involvement as SARS-CoV-2 can likely infect the GI tract via ACE 2 receptors [42]. Previous research has found throat pain to be predictor of long-term COVID [30]. We found that it was characteristic of long-term COVID in our sample population but not a predictive factor in the logistic regression analysis.

Dyspnoea has previously been reported to be associated with long-term COVID, however [30], we found no such association. It is worth noting that our cohort showed relatively lower frequencies of dyspnoea, and this could be related to the fact that we recruited only individuals who had not been hopitalised due to COVID-19.

### 4.2. Diet and Symptomology

Althoμgh the analysis of FFQ data, showed some significant differences across the symptom groups (Table 5), overall, the diets were vastly similar between the three groups. Interestingly we found the AS group consumed significantly higher amounts of poultry, while Kim et al. found that individuals who consumed diets high in vegetables and/or fish and lower in poultry had much lower chances of developing moderate-to-severe COVID-19 [12].

Nutrient intake calculated from food diaries (Table 6) revealed that except for few, dietary intake of nutrients was similar in the 3 symptom groups. The asymptomatic group consumed significantly higher vitamin E than the LC group, while the LC group consumed significantly higher amount of omega 3 and vitamin B12 than the S group but not the AS group.

Salazar-Robles et al. enrolled 103 SARS-CoV-2 positive patients and used an FFQ and measured this against symptom severity [11]. They found that increase in the intake of ‘milk and milk products’, ‘fats and oils’, ‘legumes’, ‘grains’, ‘bread’, and ‘cereals’ negatively correlated with overall symptom severity, and their regression analysis sμggested that higher consumption of legumes, grains, bread, and cereals were predictive of decreased symptom severity. In contrast we did not find significant differences of these foods between our symptom groups. We attempted to build a regression model with the same variables as this study [11], however, we were not able to predict symptom severity from these food groups in the FFQ. It was interesting to note that the analysis of nutrient intake using food diaries showed total fat and monounsaturated fat intake to be higher in the asymptomatic group than the LC group althoμgh it failed to reach a statistical significance (*p* = 0.089 and 0.057, respectively, Table 6). Fatty acids play important roles in immune function such as regulating phagocytic activity of macrophages, movement of dendritic cells into lymph nodes and activation of mast cells [43,44]. The type of fatty acids ingested can have a different effect on inflammation, saturated fats can stimulate in vivo the nod-like receptor protein (NLRP3) inflammasome, which produces pro-inflammatory cytokines IL-1β and IL-18. Unsaturated fats on the other hand have the opposite effect and inhibit the NLRP3 (NOD-, LRR- and pyrin domain-containing protein 3) inflammasome [45]. Docosahexaenoic acid (DHA) and eicosapentaenoic acid (EPA) have been shown to stimulate IgM production [46,47]. We found that the LC group consumed significantly more omega 3 than the S group but not the AS group. It is worth noting that all groups had a median N6/N3 ratio greater than 4, with the S group more than double this. Overall, the N6/N3 ratio was seen to have no effect on symptom severity in the present study.

Even thoμgh this study and the one done by Salazar-Robles et al. both used an FFQ and symptomology questionnaire to assess the relationship of diet on COVID-19 severity [11], differences in the data could be related to the study populations in question. Their study population were young individuals like present cohort (26–39 years) and had a similar ratio of female: male participants (roμghly 7:3), and all patients attended an outpatient clinic which we assume means they were also mild cases. However, their cohort had a mean BMI of 27 (considered ‘overweight’ on the BMI scale) whereas our study population had a median BMI of 23 (considered ‘healthy weight’ on the BMI scale), their population included individuals with hypertension and diabetes mellitus, known to affect the severity of COVID-19 whereas ours did not. Therefore, there is a possibility that healthy dietary habits have a greater impact in individuals who are at higher risk of severe COVID such as the overweight and underlying comorbidities known to affect COVID-19 severity.

Kim et al. documented 568 COVID-19 cases in six countries and reported that plant-based diets or pescatarian diets were associated with lower odds of moderate to severe COVID-19 [12]. In contrast we did not find any association between consumption of fish or plant-based products and COVID-19 severity. However, we were only looking at a population of mildly affected individuals, and relatively younger group and they looked at an older population (mean age 48 years). It is possible that the effect of diet becomes more prominent in older age group and population with a severe infection of SARS-CoV-2.

Merino et al. collected data from 815 COVID-19 cases and found that plant-based foods were associated with lower severity of COVID-19 [10]. They assessed severity based on participants needing to visit the hospital and if they required breathing support and medication. Once again, the effects of diet on COVID-19 severity may be more pronounced in those who needed to be hospitalised, whom we excluded from this study.

### 4.3. Food Groups and Nutrients and Symptom Severity

#### 4.3.1. Citric Fruits and Other Fruits and Fruit Juices

In this study ‘citric fruits’ positively correlated with chills severity, while the ‘other fruits’ category and ‘concentrate fruits juices’ negatively correlated with symptom severity. We also discovered that those who did not have a fever and did not have loss of smell ate more of fruits in the categories ‘other fruits’ and ‘citric fruits’ respectively. Citric fruits were labelled as: orange, mandarin other fruits were labelled as apple, pear, peach, and banana. Citric fruits typically contain high amounts of vitamin C, higher consumption of vitamin C was also associated with having fever (Table 7). Citric fruits also contain high amounts of other bioactive polyphenols such as hesperidin, narirutin and naringin to name a few, which have been reported to reduce inflammatory markers in human trials [48]. Fruit juice and fruits in general contain many antioxidants such as carotenoids, anthocyanins, and flavonoids, which counter oxidative stress and inflammation which as discussed previously, plays a significant role in COVID-19 progression [49]. One study has shown that grape juice can lower the concentration of IL-6 in rats, and naringin has been shown to inhibit SARS-CoV-2 infection in vitro [50,51]. Our results here regarding citric fruits is contradictory, other fruits (apple, pear, peach, banana) and fruit juices were associated with less disease severity here.

#### 4.3.2. Nuts

We found that individuals who consumed more nuts did not suffer from loss of smell and taste (*p* = 0.005 and 0.05, respectively, Table 7). Nuts such as almond, cashew nuts, pistachios, walnuts, and peanuts are a good source of phytochemicals; that can contribute towards lowering inflammation in the body. However, due to the lack of understanding surrounding the mechanisms involved with SARS-CoV-2 and loss/altered sense of smell/taste, we cannot propose a reason why nuts would affect these symptoms except to lower overall inflammation and disease severity.

#### 4.3.3. Protein and Energy Intake

Studies in undernourished children have shown that protein and energy malnutrition contribute to an increased risk of severe infection by reducing CD8+ T cell responses [52,53,54]. Higher intakes of protein and energy intake have been associated with better survival in critically ill COVID-19 patients by one study [55]. We found no relation between protein and energy intake and symptom severity in our study.

#### 4.3.4. Poly-Unsaturated Fatty Acids (PUFA), Omega 3 and 6, Monounsaturated Fatty Acids (MFA), Saturated Fatty Acids (SFA) 

PUFA intake negatively correlated with many symptoms in our study. One study involving nearly 1000 plasma biobank samples concluded that higher total circulating polyunsaturated fatty acids as well as specifically omega 3 and 6 PUFAs were associated with a lower risk of developing severe COVID-19 [56]. We found a significant negative correlation (*p* ≤ 0.05) between total PUFA intake and symptoms of fatigue, myalgia, runny nose, headache as well as summed severity of symptoms, however, a negative correlation between PUFA intake and chills severity failed to reach the statistical significance (*p* = 0.097, Figure 3) Additionally, Individuals who did not have a coμgh consumed significantly higher amounts of PUFAs then those who did not (Table 8). It has been sμggested that high consumption of omega 6 can promote a pro-inflammatory state due to its pro-inflammatory derivatives, however there is some dispute on whether this is true [57]. Except for a weak negative correlation between fever severity and omega 6 (r = −0.253, *p* = 0.067), we found that althoμgh total PUFA intake significantly correlated negatively with several symptoms, however when omegas 3 and 6 were examined separately (Figure 3), there was no evidence that they can affect symptom severity or the progression to long COVID-19.

#### 4.3.5. Vitamin A (Retinol)

Vitamin A (retinol) displayed a negative correlation with several symptoms. The Mann–Whitney U analysis revealed that individuals who did not suffer a coμgh consumed significantly higher amounts of retinol than those who did (*p* = 0.013). Retinol is important for the transformation of naïve T cell to regular T-cells [58]. It has been proposed that retinol depletion may play a role in the progression to severe COVID-19 [59]. A defect in type 1 interferon synthesis in severe COVID-19 patients has been observed, and due to the role of retinol in the synthesis of type 1 interferon and the fact that severe COVID-19 patents have been observed to have retinol depletion [59], many of the widespread symptoms of COVID-19 could be explained at least in part due to retinol depletion, however this is a novel hypothesis and should be investigated further [59].

#### 4.3.6. Vitamins B1, B2, B6, B9 and B12

We found no evidence to sμggest that vitamins B2, B6 and B12 play a protective role in COVID-19 severity or progression to long COVID. The LC group consumed significantly more vitamin B12 than the S group but not the AS group, however most individuals were seen to consume adequate amount of vitamin B12 (>4 μg/d) (Table 6). Higher intake of thiamine and folic acid were observed in individuals who did not suffer GI symptoms (Table 7 and Table 8). Vitamin B1 (thiamine) has been shown to be beneficial in patients with inflammatory bowel disease and folate deficiency has been associated with the development of inflammatory bowel disease [60]. Thiamine has been used as an adjuvant therapy in a two-centre COVID study and was associated with lower incidence of thrombosis [61]. Folate is vital for the processes involved in DNA and protein synthesis. The epithelium of the gastrointestinal tract has the highest turnover rate of any tissue in the body, this turnover rate may even increase during COVID-19 infection, as a result of SARS-CoV-2 entry into GI cells via ACE 2 [42]. Folic acid has even been sμggested to be used as an adjuvant therapy in the early stages of COVID-19 as it is sμggested to prevent binding of the SARS-CoV-2 spike protein, thoμgh further studies are needed to confirm these findings [62]. To our knowledge, the present study is the first to associate folate and thiamine with GI symptom severity in COVID-19.

Folic acid deficiency anaemia leads to poor oxygen transport, which has been shown in the case of COVID-19 to be related to the development of dysgeusia [63]. We observed a weak negative correlation (r = −0.245) with folate and dysgeusia severity, althoμgh statistical significance was not achieved (*p* = 0.077).

#### 4.3.7. Vitamin C

Several weak positive correlations were seen between vitamin C and symptom severity, however none of them reached significance. The *t*-test (Table 7) showed that prevalence of fever was higher in individuals with high intake of vitamin C. Fever is a natural response to viral infection, inflammation and an increase in body temperature are a natural consequence of this. Systematic review and meta-analysis support the use of high dose vitamin C in the prevention of respiratory infections due to the modulatory effects of vitamin C on the production of inflammatory markers caused by viral infection [64,65]. A previous study has reported that ingestion of high dose vitamin C in healthy individuals raises the body temperature 2 h post ingestion [66]. Interestingly, vitamin C has also been reported to enhance the resistance to cold by raising the body temperature and the basal metabolism rate [67].

Chills are defined as involuntary muscle contractions in response to the body feeling cold. Chills are typically a predictor of fever and as such, it is interesting that ‘citric fruits’ showed a mid-strength positive correlation with chills severity while ‘concentrate fruit juices’ showed a slight negative correlation with chills severity (Figure 3). Citric fruits are one of the best sources of vitamin C, however, concentrate fruit juices may or may not contain high levels of vitamin C depending on the type of juice that is consumed. Vitamin C has been shown in systematic review and meta-analysis to decrease pro-inflammatory cytokines such as IL-6. Citric fruits typically contain high amounts of vitamin C as well as other bioactive polyphenols such as hesperidin, narirutin and naringin to name a few, which have been reported to reduce inflammatory markers in human trials [48]. One recent systematic review and meta-analysis concluded that there was no significant benefit of vitamin C administration for the treatment of COVID-19, however randomised control trails (RCTs) are likely to be a better study design to address this question [68].

#### 4.3.8. Vitamin D

We found no evidence that dietary intake of vitamin D has any influence on the severity of symptoms or progression to long COVID in this cohort. In general individuals were consuming much less vitamin D in the diet than the recommended value (Table 6), and this is reflected in the serum concentrations shown in Table 9. Meta-analysis has demonstrated that there is still insufficient evidence to conclude whether vitamin D does or does not play a role in COVID-19 severity [69]. Our preliminary evidence sμggests that at least vitamin D does not play a role in symptom severity in young individuals with mild infection of COVID-19.

#### 4.3.9. Vitamin E

Individuals with a higher intake of vitamin E showed a lower persistence/presence of GI symptoms, coμgh and throat pain, sμggesting that vitamin E may have a protective role in the severity of these symptoms.

Vitamin E negatively correlated with fatigue, myalgia, throat pain, coμgh and the summed severity experienced by individuals (Figure 3), as well as chills and number of symptoms althoμgh significance was not achieved for these two (*p* = 0.071 and 0.052 respectively). Vitamin E has been associated with decreased incidence of infection in several studies, althoμgh the magnitude of its effects have been found to be small [70]. Its supplementation has been shown to increase the antibody response in humans, as well as promote T cell proliferation and NK cell activity [70]. The recommended dietary allowance of vitamin E for males and females above 14 years of age is 15 mg daily [71]. Althoμgh average daily intake of vitamin E was lower than the recommended intake in our participants (Table 6), the LC participants had significantly lower intake of vitamin E than the asymptomatic group but not the symptomatic group. This implies vitamin E may play a protective role in progression to long COVID-19. Vitamin E supplementation could be beneficial for vitamin E deficient individuals with COVID-19 or possibly even other infectious disease. However, it is first necessary to investigate this relationship further.

#### 4.3.10. Iron

Iron consumption was found to negatively correlate with myalgia, loss of taste and summed severity. Additionally, compared to individuals who suffered GI symptoms, iron intake was found to be higher in individuals who did not suffer GI symptoms (Table 7). Some viruses have been shown to change the metabolism of iron to support their growth via influencing human haemochromatosis protein or hepcidin [72]. This can lead to an increase in unbound iron which, due to its potential to generate oxidative stress, can promote a decline in the already diminished health of the host. Previous research has demonstrated a U-shaped relationship between serum iron levels and COVID-19 severity [73]. Iron is necessary for the immune cell proliferation, and receptors for iron transport protein transferrin are expressed on many immune cells (monocytes, macrophages, and T cells) [74]. Systematic review and meta-analysis have revealed that COVID-19 patients have issues with iron homeostasis, typically presenting with hyperferritinaemia (it is worth noting, ferritin is an acute phase protein and levels typically increase during infection) and decreased haemoglobin concentration indicative of anaemia, both of which have been shown to be markers of COVID-19 severity [75]. Iron supplementation can therefore be considered for COVID-19 patients who present with disturbances in iron homeostasis and GI symptoms.

#### 4.3.11. Copper

We found copper correlated to dysgeusia severity and those who reported dysgeusia consumed significantly less copper. Several mechanisms have been proposed for the development of loss/altered sense of taste. These include: direct infection of taste buds and salivary glands via the ACE 2 receptor, direct damage to the cranial nerves responsible for gustation, inflammation of the oral mucosa, tissue hypoxia and acute hypozincaemia [63].

Copper intake has been shown to have both pro-oxidant and antioxidant effects, and therefore its role in inflammatory processes is still somewhat unclear [76]. Copper has been linked to patient survival and has been proposed as a possible adjuvant therapy for COVID-19 [77,78]. Due to a lack of certain understanding about how dysgeusia occurs in COVID-19 patients, we do not propose a specific mechanism throμgh which copper could influence dysgeusia, thoμgh this should be investigated further.

#### 4.3.12. Selenium

We found no evidence to support the role of selenium in COVID-19 severity or progression to long COVID. Selenium plays a role in both inflammatory and immune responses, as well as thyroid hormone metabolism. Selenium deficiency has been linked to worse outcomes in COVID-19 [79]. In our cohort, both the AS group and S group consumed a lower amount of selenium compared to the LC group, however, the levels consumed were approximately 73% of the recommended value which sμggests our participants mostly had an adequate intake of selenium.

#### 4.3.13. Zinc

We would firstly like to point out that no relation was seen between zinc consumption and loss of smell and taste. We mention this particular result because zinc insufficiency has been sμggested to play a role in smell and taste disturbances [80].

We found that individuals who did not suffer GI symptoms consumed significantly higher amounts of zinc than those who did (Table 7). Zinc plays an important role in cell mediated immunity, as well as being an antioxidant and anti-inflammatory agent in the body. Evidence from in vitro studies sμggests that SARS-CoV-2 replication can be inhibited by increase in zinc concentration [81]. Zinc supplementation studies have shown decreased oxidative stress due to its participation in free radical scavenging and pro-inflammatory markers levels such as IL-1, IL-6 and TNFα, even during infection [82,83]. Zinc may therefore reduce the inflammation in the gut caused by SARS-CoV-2. One RCT concluded that high dose zinc supplementation does not affect SARS-CoV-2 symptoms, however severity of GI symptoms was not measured in this study [84], and we propose that more research be carried out to examine if zinc plays a protective role in the severity of GI symptoms in COVID-19.

#### 4.3.14. Fibre

We found that individuals who consumed higher amount of fibre were less likely to suffer GI symptoms (Table 8). As mentioned previously, the SARS-CoV-2 virus enters cells of the large intestine via the ACE 2 receptor [42]. This has been observed to cause intestinal cell damage which may play a role in causing some of the symptoms associated with the GI tract during SARS-CoV-2 infection such as diarrhoea [85]. Microbiota composition has been shown to be significantly altered in patients with COVID-19 and this altered microbiota composition has been associated with worse COVID-19 disease severity in several studies [86]. One study used a high fibre formula intervention to alleviate long-term COVID symptoms with considerable success, however the intervention was only used on one patient with no control group [87]. A high fibre diet to relieve COVID-19 symptoms has been sμggested but not yet been tested thoroμghly in a clinical setting. We propose fibre can also be considered as an adjuvant therapy for individuals with GI symptoms in COVID-19.

### 4.4. Serum Vitamin D Concentration and COVID-19 Severity

Our second aim was to find out if serum 25(OH)D concentrations were different in individuals with long COVID compared to the asymptomatic and mildly symptomatic groups. It is worth noting that serum levels of 25(OH)D in our cohort was in the insufficient range for all groups (Table 9). In contrast with previous studies untaken in older age group, where reduced serum vitamin D levels have been associated with severity of COVID-19, this has not been seen in the young age group recruited in the present study. Future studies may want to examine if 25(OH)D supplementation has any effect on disease severity in young and healthy individuals.

### 4.5. Immunological Analysis

With respect to the SARS-CoV-2 spike IgG antibody response, we observed antibody responses for a maximum time of 479 days after infection. The strongest responses were seen in the first 300 days of infection, which the strongest being 502 BAU/mL at 92 days after infection. Long-term COVID individuals had significantly higher IgG concentrations than the asymptomatic and mildly symptomatic individuals (Table A4b). Imai et al. analysed 611 serum samples and found that individuals with more severe COVID-19 generate higher IgG responses [88]. We also found however that the younger individuals tended to have higher amounts of SARS-CoV-2 antibodies. Klein et al. analysed over 100 positive antibody samples and found that older individuals have higher concentrations of IgG antibodies, however they analysed a much bigger age range than ours, all the individuals in our study could be considered relatively young, and yet our data sμggests that even between <25 and 36–50 years there can be significant differences in the concentrations of spike IgG (Table A4a) [89].

In the cross-sectional antibody analysis, 33 samples were above the threshold for positivity. The median period for which antibodies were detectable was 296 (85 to 479), days for the AS group, 94 (42 to 264) days for the S group and 110 (27 to 465) days for the LC group.

The longitudinal antibody analysis revealed an increase in antibody titre of two individuals between months one and nine post infection, where the serum concentration of spike IgG multiplied by 3–5 times over a 6–8-month period. Dan et al. also performed longitudinal analysis for IgG spike protein for 51 individuals over 8 months and found an average half-life of 103 days for spike IgG [90]. After examining their data, we could find no instance where the spike IgG antibody concentration continued to increase for as long or as drastically as we saw with the two participants in our study cohort. We decided to examine these two cases in detail to look for differences in their diet and lifestyle compared to the other 10 participants, Table A5 However, none of dietary and lifestyle habits stood out different in these two participants except for the supplements they took. One participant (labelled N in Figure 5) was taking daily supplements of Complidermol 5 Alfa plus; a mixture of B vitamins, selenium, iron, zinc, and herbal extracts; Pygeum Africanum, Serenoa Repens and Dimetil Sulfona. Second participant who also showed a steady increase in antibody levels (labelled T in Figure 5) reported taking daily supplements of C. sinensis, a ascomycetes fungus. Whether these supplements had any effect on their increased antibody response is impossible to ascertain at this time, and more studies are needed to examine the effects of supplement intake on COVID-19 severity and immune response.

Food groups from the FFQ were correlated to spike IgG titres as shown in Table 10. Natural fruit juices correlated negatively with antibody concentration while other fruits (this included fruits such as apple, pear, peach, and banana) correlated positively with antibody titres. Natural fruit juices are a good source of nutrients such as vitamin C, flavanones, and folate, all of which have been shown to support the function of immune cells. Hesperidin, a flavanone found particularly high in citrus fruits has been reported to interfere with the entry of SARS-CoV-2 into host cells and inhibit viral replication in vitro [91]. As mentioned previously, naringenin, a flavonoid found in citrus fruits has also been found to have a strong anti-viral activity against SARS-CoV-2 [51]. Citrus fruit intake, however, did not show a significant correlation with antibody response in our study. We asked participants which type of natural fruit juice they typically consumed, and, of the 27 participants who consumed natural fruit juices, only 18 responded and 14 mentioned “orange”, 3 responded “orange and lemon”, and, one responded “banana, orange, mango, and apple”. Natural fruit juices provide a concentrated source of nutrients; therefore, effects of their nutrients can be higher than of the fruit itself. However, it is worth noting that antiviral properties reported against SARS-CoV-2 for both hesperidin and naringenin were shown for the in vitro studies, therefore, further research is needed to examine if findings of in vitro studies can be replicated in vivo.

### 4.6. Limitations

This study does have few limitations, primarily the small sample size. Another limitation is the timing of the blood sample collection post infection was not consistent. The reason being that individuals were invited to complete the questionnaires and provide their blood samples following the positive PCR test. Differences in antibody concentrations between groups may have been different if all individuals provided their blood samples precisely at a fixed time, i.e., one- and six-months post infection.

The FFQ completed by participants reported the dietary habits of individuals pre-infection with COVID-19. This relies on participants accurately remembering what they were eating at the time (AKA recall bias). It is therefore possible that some of the data was under or over-reported. For the food diaries, dietary intake of nutrients was recorded post-COVID-19. It is possible that this group made changes to their dietary habits after, COVID-19, especially those experiencing symptoms of long-term COVID. However, all questionnaires were subject to the bias relating to self-reported data which can also lead to under-reporting or over-reporting of data.

At the time that this study began (January 2021), there was no published clinical definition for Long COVID. So, we asked participants to define themselves on the basis of if they suffered recurrent COVID-19 symptoms after having tested negative for COVID-19. Some diligence should therefore be taken when comparing results from our long COVID individuals to other data, depending on how long COVID was defined in those studies.

Among the strengths of our study is the longitudinal collection and analysis of data from individuals before COVID-19 vaccination, in combination with the assessment of serum immunoglobulin and 25(OH)D levels and the assessment of diet via two dietary collection methods.

## 5. Conclusions

In conclusion, our data indicates that fatigue and number of symptoms reported are predictive factors of long-term COVID and that rhinorrhoea is a predictor of not developing long-term COVID. To the best of our knowledge, we are the first to provide evidence that rhinorrhoea could be considered as a predictive factor of not developing long-term COVID, despite this however, some LC individuals did experience rhinorrhoea in this study, therefore, this warrants further investigations in more powered studies.

Our results show that COVID-19 IgG antibodies lasted for more than a year in some participants but declined quickly for others. Similar trends have been seen and it remains unclear why only some individuals develop a sustained IgG response after infection [24,92,93,94,95]. We have not been able to clearly identify any food groups or nutrients that could affect the longevity of antibodies in the blood.

This study did not find any difference between serum 25(OH)D levels of individuals with and without long COVID. Other studies have shown a relation between vitamin D levels and symptom severity [19,21]. However, a recent meta-analysis has concluded that there is still insufficient evidence to conclude whether vitamin D does or does not play a role in COVID-19 severity [69].

In this study we did find evidence that several nutrients may play a role in severity and progression to long COVID. The strongest evidence was for the role of poly-unsaturated fatty acids, vitamin E, iron, and fibre in the diet, with some evidence for a possible role of nutrients such as zinc, folate, and retinol. We believe PUFA, iron, fibre and vitamin E should be considered further in relation to their possible protective role in COVID-19 disease especially vitamin E which showed several significant negative correlations with symptom severity (Figure 3), and significantly lower intake was seen in those with long COVID-19 (Table 6).

Althoμgh this is a pilot study, the data provided sμggests casual relations that should be examined further with more powered studies. Althoμgh it seems SARS-CoV-2 does not pose a big threat now as when the pandemic began, understanding factors that can affect its severity can help us prepare for future disease outbreaks.

## Figures and Tables

**Table 1:** Grouping of participants base on the severity of disease.

Group 1 (AS)	Group 2 (S)	Group 3 (LC)
Asymptomatic/without symptoms	Mild/Moderately Symptomatic with no long-term COVID	Mild/moderately Symptomatic with long-term COVID

**Table 2:** Characteristics of the study cohort.

	Total Cohort (N = 55)	AS Group (n = 15)	S Group (n = 15)	LC Group (n = 25)
Age (Years)	32 (21 to 50)	35 (23 to 48)	29 (24 to 45)	33 (21 to 50)
Gender				
Male	15 (27%)	2 (13%)	6 (40%)	7 (28%)
Female	40 (73%)	13 (87%)	9 (60%)	18 (72%)
BMI	22.8 (19.1 to 35.7)	23.2 (19.1 to 32.8)	22.3 (19.2 to 31.6)	23.1 (19.2 to 35.7)
Smoker status				
Current smoker	9 (16%)	3 (20%)	2 (13%)	4 (16%)
Previous smoker	10 (18%)	2 (13%)	2 (13%)	6 (24%)
Never smoked	36 (66%)	10 (67%)	11 (74%)	15 (60%)
Questionnaires and Food diary				
Both questionnaires and food diary completed	53 (96%)	14 (93%)	14 (93%)	25 (100%)
FFQ + Food diary uncompleted	2 (4%)	1 (7%)	1 (7%)	0 (0%)
Symptomology uncompleted	0 (0%)	0 (0%)	0 (0%)	0 (0%)
Number of participants that provided blood samples	52 (95%)	15 (27%)	13 (24%)	24 (44%)
Two time point donors	39 (75%)	11 (73%)	9 (69%)	19 (79%)
Single time point donors	13 (25%)	4 (27%)	4 (31%)	5 (21%)

Data are shown as absolute value (percentage) or for age and BMI, median (range) values are given. Where percentages were given as decimals they have been rounded to the nearest whole number. BMI—body mass index.

**Table 3:** Chi square analysis of different symptoms experienced by the mild symptom (S) and long-term COVID patients (LC).

	X2(1)	*p*	BF01	OR
Fatigue	N/A	0.008	19.23	16.75
Throat pain	7.11	0.008	12.99	8.23
Runny nose	4.00	0.046	2.52	0.26
Nausea/vomiting	N/A	0.033	5.85	N/A
Diarrhoea	N/A	0.016	9.80	N/A

X2(1) = Pearson’s chi square statistic; BF01 = Bayes factor, OR = Odds ratio (long-term COVID/symptomatic). Note, where no chi square statistic was shown, fisher’s exact test was used due to low frequency reporting of a symptom. OR could not be calculated when frequencies were 0 for a particular cell.

**Table 4:** Coefficients of the model predicting which symptomatic individuals developed long-term COVID (95% BCa bootstrap confidence intervals based on 1000 samples in brackets).

	B	95% CL for Odds Ratio		
		Lower	Odds Ratio	Upper
Variables Included				
Constant	−3.95			
Fatigue	2.05 [0.13, 22.63]	0.61	7.73	97.74
Number of symptoms experienced	0.63 [0.25, 2.11]	1.15	1.88	3.07
Rhinorrhoea (Runny nose)	−2.41 [−26.7, −1.46]	0.01	0.09	0.75

B is a logistic regression coefficient Note. R2 = 0.67 (Hosmer-Lemeshow), 0.43 (Cox-Snell), 0.58 (Nagelkerke). Model X2(1) = 21.89 *p* < 0.0001.

**Table 5:** FFQ analysis comparison across the 3 symptom groups.

Food Group	Asymptomatic	Symptomatic	Long-Term COVID
Chicken or turkey	2.5 ^a^ (0.0–7.0), 3.3	2.0 ^ab^ (0.0–4.5), 2.0	1.0 ^b^ (0.0–14.0), 2.0
Cream or chocolate cakes	0.4 ^a^ (0.0–7.0), 1.0	0.2 ^ab^ (0.0–1.0), 0.3	0.0 ^b^ (0.0–1.3), 0.3
Sweets	0.0 ^ab^ (0.0–1.3), 0.6	0.4 ^a^ (0.0–1.0), 0.8	0.0 ^b^ (0.0–1.0), 0.3
Ice cream	0.3 ^ab^ (0.0–2.0), 1.0	0.6 ^a^ (0.0–3.0), 0.8	0.0 ^b^ (0.0–2.0), 0.8
White rice, paella	1.0 ^ab^ (0.5–3.5), 1.0	1.5 ^a^ (0.7–4.5), 2.1	1.0 ^b^ (0.0–14.0), 0.6

Data are reported as portions/week, showing the median (range), IQR. Those not sharing same superscript within a row were significantly different at *p* ≤ 0.05 in the Mann–Whitney U test.

**Table 6:** Analysis of the food diary showing a comparison of specific nutrient intake among patients grouped based on their symptoms.

	AS Group	S Group	LC Group	DRV for Adults
Energy (Kcal)	1541 (886–2387), 439	1402 (935–2281), 561	1538 (749–3059), 572	(M) 2550 (F) 1940
Carbohydrate (g)	137 (71–216), 58	126 (82–267), 82	128 (46–303), 79	45–60 E%
Protein (g)	71 (45–119), 15	75 (48–102), 27	83 (55–201), 29	0.66–0.83 g/kg body wt/per day
Fat (g)	72 (32–122), 20	69 (33–97), 28	65 (34–100), 17	20–35 E%
SFA (g)	22 (9–40), 13	18 (8–32), 15	18 (8–43), 9	
MUFA fat (g)	28 (8–46), 17	27 (5–45), 22	19 (7–47), 12	
PUFA (g)	11 (3–29), 10	8 (5–16), 6	8 (3–18), 4	
PUFA/SFA ratio	0.5 (0.2–1.3), 0.5	0.5 (0.2–1.1), 0.3	0.5 (0.2–1.1), 0.3	
Omega 3 (g)	**0.5 ^ab^** (0.1–2.7), 0.9	**0.3 ^a^** (0.0–1.4), 0.4	**0.7 ^b^** (0.1–2.7), 0.8	
Omega 6 (g)	2.6 (0.6–6.6), 3.1	2.5 (0.2–5.2), 3.2	1.9 (0.5–6.3), 2.7	
ω6/ω3 ratio	**5.5 ^ab^** (1.1–14.6), 6.2	10.5 ^a^ (1.8–17.7), 9.7	**4.1 ^b^** (0.3–8.6), 3.9	4:1
Fibre (g)	17.1 (10.3–31.4), 6.2	15.9 (9.5–31.6), 8.8	14.1 (6.9–33.6), 7.1	25 g/d
Alcohol (g)	2.7 (0.0–19.8), 9.6	1.1 (0.0–22.0), 5.7	0.0 (0.0–42.2), 6.5	
Iron (mg)	10.8 (5.2–23.8), 3.8	8.2 (5.0–15.6), 2.9	8.1 (5.4–22.6), 5.2	(M) 6 mg/d (F) 7 mg/d
Zinc (mg)	7.1 (4.4–11.9), 3.8	6.0 (3.1–10.3), 3.0	6.7 (4.0–17.5), 4.4	(M) 7.5–12.7 mg/d (F) 6.2–10.2 mg/d
Selenium (μg)	51 (27–89), 20	56 (25–102), 43	70 (26–210), 46	70 μg/d
Copper (mg)	0.8 (0.3–1.3), 0.7	0.7 (0.2–1.4), 0.4	0.7 (0.3–2.1), 0.7	(M) 1.6 mg/d (F) 1.3 mg/d
Vitamin A (μg)	642 (426–1443), 219	511 (138–983), 270	549 (237–1978), 659	(M) 900 μg/d (F) 700 μg/d
Vitamin D (μg)	2.0 (0.3–14.6), 4.9	3.7 (1.0–14.1), 5.6	4.1 (0.6–15.2), 3.8	15 μg/d
Vitamin E (mg)	**8.6 ^a^** (2.5–16.0), 7.2	**7.4 ^ab^** (3.2–14.7), 6.0	**6.2 ^b^** (2.7–18.7), 3.8	(M) 13 mg/d (F) 11 mg/d
Vitamin K 1 (μg)	33.4 (5.1–141.2), 60.9	39.1 (5.9–114), 68.8	27.6 (4.7–127.0), 36.1	(M) 120 μg/d (F) 90 μg/d
Thiamin (mg)	1.1 (0.7–2.1), 0.7	1.2 (0.7–9.7), 0.5	1.2 (0.6–2.7), 0.7	(M) 1.2 mg/d (F) 1.1 mg/d
Riboflavin (mg)	1.2 (0.6–2.6), 0.6	1.1 (0.5–1.4), 0.5	1.3 (0.5–2.4), 0.7	1.3 mg/d
Niacin (mg)	28.6 (16.6–75.4), 11.0	26.6 (12.8–42.2), 18.8	33.6 (13.5–92.4), 19.0	(M) 16 mg/d (F) 14 mg/d
Tryptophan (mg)	606 (114–1010), 410	514 (139–1030), 324	640 (142–2419), 477	250–425 mg/d1
Vitamin B6 (mg)	1.8 (0.7–2.9), 1.2	1.5 (0.8–2.8), 1.1	1.7 (0.9–3.8), 0.7	(M) 1.5 mg/d (F) 1.3 mg/d
Folate (μg)	212 (132–319), 68	195 (84–345), 90	185 (97–513), 131	250 μg/d
Vitamin B12 (μg)	**4.5 ^ab^** (1.0–12.7), 3.1	**3.5 ^a^** (1.0–7.1), 3.5	**5.4 ^b^** (2.0–19.2), 5.1	4 μg/d
Biotin (μg)	19.5 (5.7–78.5), 21.1	16.4 (5.4–32.5), 13.0	22.0 (6.4–69.4), 13.6	40 μg/d
Vitamin C (mg)	74 (31–193), 69	80 (15–264), 59	87 (23–376), 56	(M) 90 mg/d (F) 80 mg/d
Iodine (μg)	100 (28–150), 67	76 (19–151), 53	94 (33–185), 71	150 μg/d

Values are shown as median (range), IQR. Median values of nutrients not sharing same superscript within a row (highlighted in bold) were significantly different at *p* ≤ 0.05 in the Mann–Whitney U test. PUFA—Poly-Unsaturated Fatty Acids; SFA—Saturated Fatty Acids; MUFA—Monounsaturated Fatty Acids; DRV—Daily Recommended Values, (M) male (F) female. Daily reference intakes are shown for adults above the age of 18 years but below menopausal age and not applicable for the pregnant, according to the European Food Safety Authority EFSA, except for tryptophan which was taken from Richard et al. [29].

**Table 7:** Differences in mean consumption of food groups/nutrients that were significantly different across individuals who did or did not experience a particular symptom in the independent *t*-test.

	Symptom/s	‘Yes’ Mean (SD)	‘No’ Mean (SD)
*FFQ (p/w)*			
Citric fruits	Loss of sense of smell	1.8 (2.2)	* 3.8 (4.4)
Other fruits	Fever	3.5 (3.1)	* 7.5 (6.0)
Nuts	Loss of sense of smell Loss of sense of taste	2.0 (2.8) 2.0 (1.0)	*** 3.6 (2.9) * 3.5 (2.8)
*Food diary*			
Energy (Kcal)	Rhinorrhoea, GI symptoms	1327 (358) 1287 (316)	* 1564 (428) * 1559 (430)
Carbohydrate (g)	Rhinorrhoea, Loss of sense of taste	114 (47) 125 (59)	* 147 (51) * 147 (43)
Saturated fat (g)	Rhinorrhoea	16.1 (7.3)	* 20.1 (7.3)
Fibre (g)	Loss of sense of taste	14.8 (6.4)	* 17.7 (5.6)
Vitamin C (mg)	Fever	117.4 (86.6)	* 78.0 (45.6)
Vitamin E (mg)	GI symptoms	5.8 (2.3)	* 8.2 (3.8)
Folates (mg)	GI symptoms	160 (38)	* 225 (93)
Iron (mg)	GI symptoms	7.0 (1.5)	*** 10.4 (4.3)
Zinc (mg)	GI symptoms	6.1 (1.2)	* 7.6 (2.8)
Sodium (mg)	GI symptoms	1243 (397)	** 1814 (750)

Note. Data is presented as mean (SD) because the data was transformed using Log10 to allow for the use of parametric *t*-test. All Significance is shown between those who suffered a symptom(s) and those who did not. GI symptoms (nausea/vomiting, diarrhoea and/or abdominal pain), Other fruits: ‘apple, pear, peach, banana’. *p* ≤ 0.05 *, ≤0.01 **, ≤0.005 *** *p*/*w*—Portions per week.

**Table 8:** Differences in the consumption of food groups/nutrients that were significantly different across individuals who mentioned ‘Yes’ or ‘No’ to experience of a particular symptom, significance was determined using Mann–Whitney U tests.

	Symptom/s	‘Yes’	‘No’
*Food diary*			
Carbohydrate (g)	Rhinorrhoea	108 (46–216), 73	* 141 (60–303), 69
Saturated fat (g)	Coμgh	14.3 (8.0–42.7), 11.4	* 19.7 (8.4–40.4), 9.8
Poly-unsaturated fat (g)	Rhinorrhoea	7.0 (4.2–13.8), 4.1	* 9.0 (3.2–29.2), 6.8
Monounsaturated fat (g)	Chills	20.6 (5.0–47.0), 13.6	* 27.1 (7.2–46.2), 20.0
	Fatigue	17.8 (5.0–44.6), 15.9	* 28.1 (7.7–47.0), 17.1
	Coμgh	15.1 (5.0–47.0), 15.4	* 26.1 (7.4–46.2), 17.4
	Rhinorrhoea	15.5 (5.0–38.8), 15.1	* 25.1 (6.5–47.0), 17.0
	GI symptoms	14.5 (6.5–36.5), 15.7	* 25.7 (5.0–47.0), 17.9
Fibre (g)	GI symptoms	13.6 (8.4–19.3), 4.2	* 16.0 (6.9–33.6), 7.4
Retinol (μg)	Coμgh	118 (0–666), 90	* 171 (37–496), 155
Vitamin E (mg)	Throat pain, Coμgh	5.3 (2.7–18.7), 3.5 5.3 (3.2–18.7), 4.1	* 8.0 (2.5–16.0) 5.2 * 7.7 (2.5–16.0), 5.2
Thiamine (mg)	GI symptoms	0.9 (0.5–1.3), 0.5	*** 1.2 (0.7–9.7) 0.7
Copper(mg)	Loss of sense of taste	0.6 (0.2–2.1), 0.4	* 0.9 (0.3–1.5), 0.4

Note. Results shown are Median (range), IQR values for nutrient intake comparison between participants who answered “Yes” or “No” to particular symptoms. * *p* ≤ 0.05, *** *p* ≤ 0.005.

**Table 9:** Serum Vitamin D concentrations across the 3 symptom groups.

	Serum 25(OH)D Levels ng/mL Median (Range), IQR
Asymptomatic n = 15	22.0 (12.4–33.7), 12.3
Symptomatic n = 13	22.4 (14.4–32.0), 7.5
Long COVID n = 24	24.9 (12.1–51.6), 9.4

Some participants (n = 39) provided their blood samples twice, i.e., once between December and April 2021, and second time in July 2021. Average of vitamin D levels were taken for the participants who provided their blood samples twice: Deficiency levels of vitamin D are <10 ng/mL, insufficiency 10–30 ng/mL, sufficiency 30–100 ng/mL.

**Table 10:** Correlation coefficients and significance values for semi partial correlations of antibody levels with weekly portions of fruits and fruit juices analysis from FFQ.

	Citric Fruits	Other Fruits	Natural Fruit Juices
Whole group			
r	0.107	0.289 *	−0.259
*p*	0.437	0.032	0.056
Asymptomatic			
r	0.079	0.489 *	−0.478 *
*p*	0.747	0.034	0.038
Symptomatic			
r	−0.049	0.027	−0.240
*p*	0.868	0.928	0.409
Long-term COVID			
r	0.271	0.412	−0.098
*p*	0.234	0.063	0.671

Correlation coefficients and significance values for semi partial correlations of weekly portions of citric fruits, other fruits, and natural fruit juices with antibody concentrations, controlling for age and time after infection. FFQ- Food Frequency Questionnaire, r = correlation coefficient with Spike IgG concentration, *p* = significance * ≤0.05, examples of citric fruits given in FFQ: oranges, mandarins, examples of other fruits given in FFQ: apple, pear, peach, banana.

## Data Availability

The data presented in this study are available on request from Kaine M McDaid. Data is not publicly available due to privacy and confidentiality issues; requests for data must be approved by the Ethics Committee after the presentation of a study protocol.

## Appendix A

**Table A1:** Symptomology Questionnaire.

Unique ID Code:	
Data Collector Information	
Name of data collector	
Email	
Form completion date (DD/MM/YYYY)	__/__/____
Participant information	
Sex	Man □ Woman □ Other □
Date of birth (DD/MM/YYYY)	
Age	
Weight (kg)	
Height (m)	
Average hours slept per night	
Blood group	
Do you use supplements? Please provide details of the ingredients/brand, how much and how often you take them.	
Do you take any medicines? Please provide details of the brand, how much and how often you take them.	
Are you a smoker?	Yes □ No □ If yes, how long-term have you smoked for?
Are you an ex-smoker?	Yes □ No □ If yes, how long-term have you not smoked for?
Country of residence	
Nationality	
Ethnicity (optional)	
Please tell us when you have had a positive result for a COVID-19 test at the university (PCR, antigen, antibody)	Yes □ No □ Unknown □ If yes, date of test (DD/MM/YYYY): __/__/____ In case of multiple positive results, please list all dates:
Have you had a negative result on a COVID-19 test? (PCR, antigen, antibody)	Yes □ No □ Unknown □ If yes, date of test (DD/MM/YYYY): __/__/____ In case of multiple negative results, please list all dates:
Have you received one or more doses of a COVID-19 vaccine?	Yes □ No □ If yes, date of first dose (DD/MM/YYYY): __/__/____ Please state which brand of vaccine you received (if known):
Symptom history	
Did you suffer any symptoms due to a COVID-19 infection?	Yes □ No □ Date that symptoms started (DD/MM/YYYY): __/__/____ Date that symptoms ended (leave blank if symptoms are ongoing) (DD/MM/YYYY): __/__/____
Have you suffered any recurring or long-term symptoms after being infected with COVID-19?	Yes □ No □
Date that the COVID-19 infection started (approximately) (DD/MM/YYYY): __/__/____ Date that the COVID-19 infection ended (leave blank if infection is ongoing) (DD/MM/YYYY): __/__/____	
Below is a list of symptoms. Please indicate which symptoms you have suffered due to a COVID-19 infection. Please list how severe each symptom was and the duration of each symptom in days from when the symptom began until end	
Fever ≥ 38 °C	Yes □ Severity (Low/Medium/High) No □ Date that this symptom started (DD/MM/YYYY): __/__/____ Date that this symptom ended (leave in blank if symptom is ongoing) (DD/MM/YYYY): __/__ ____ Duration of symptom (days):
Chills	Yes □ Severity (Low/Medium/High) No □ Date that this symptom started (DD/MM/YYYY): __/__/____ Date that this symptom ended (leave in blank if symptom is ongoing) (DD/MM/YYYY): __/__/____ Duration of symptom (days):
Fatigue	Yes □ Severity (Low/Medium/High) No □ Date that this symptom started (DD/MM/YYYY): __/__/____ Date that this symptom ended (leave in blank if symptom is ongoing) (DD/MM/YYYY): __/__/____ Duration of symptom (days):
Muscular pain (Myalgia)	Yes □ Severity (Low/Medium/High) No □ Date that this symptom started (DD/MM/YYYY): __/__/____ Date that this symptom ended (leave in blank if symptom is ongoing) (DD/MM/YYYY): __/__/____ Duration of symptom (days):
Sore throat	Yes □ Severity (Low/Medium/High) No □ Date that this symptom started (DD/MM/YYYY): __/__/____ Date that this symptom ended (leave in blank if symptom is ongoing) (DD/MM/YYYY): __/__/____ Duration of symptom (days):
Coμgh	Yes □ Severity (Low/Medium/High) No □ Date that this symptom started (DD/MM/YYYY): __/__/____ Date that this symptom ended (leave in blank if symptom is ongoing) (DD/MM/YYYY): __/__/____ Duration of symptom (days):
Runny nose (rhinorrhea)	Yes □ Severity (Low/Medium/High) No □ Date that this symptom started (DD/MM/YYYY): __/__/____ Date that this symptom ended (leave in blank if symptom is ongoing) (DD/MM/YYYY): __/__/____ Duration of symptom (days):
Breathing difficulties (disnea)	Yes □ Severity (Low/Medium/High) No □ Date that this symptom started (DD/MM/YYYY): __/__/____ Date that this symptom ended (leave in blank if symptom is ongoing) (DD/MM/YYYY): __/__/____ Duration of symptom (days):
Wheezing	Yes □ Severity (Low/Medium/High) No □ Date that this symptom started (DD/MM/YYYY): __/__/____ Date that this symptom ended (leave in blank if symptom is ongoing) (DD/MM/YYYY): __/__/____ Duration of symptom (days):
Chest pain	Yes □ Severity (Low/Medium/High) No □ Date that this symptom started (DD/MM/YYYY): __/__/____ Date that this symptom ended (leave in blank if symptom is ongoing) (DD/MM/YYYY): __/__/____ Duration of symptom (days):
Other respiratory symptoms	Yes □ Severity (Low/Medium/High) No □ Date that this symptom started (DD/MM/YYYY): __/__/____ Date that this symptom ended (leave in blank if symptom is ongoing) (DD/MM/YYYY): __/__/____ Duration of symptom (days):
Headache	Yes □ Severity (Low/Medium/High) No □ Date that this symptom started (DD/MM/YYYY): __/__/____ Date that this symptom ended (leave in blank if symptom is ongoing) (DD/MM/YYYY): __/__/____ Duration of symptom (days):
Nausea/vomiting	Yes □ Severity (Low/Medium/High) No □ Date that this symptom started (DD/MM/YYYY): __/__/____ Date that this symptom ended (leave in blank if symptom is ongoing) (DD/MM/YYYY): __/__/____ Duration of symptom (days):
Abdominal pain	Yes □ Severity (Low/Medium/High) No □ Date that this symptom started (DD/MM/YYYY): __/__/____ Date that this symptom ended (leave in blank if symptom is ongoing) (DD/MM/YYYY): __/__/____ Duration of symptom (days):
Diarrohea	Yes □ Severity (Low/Medium/High) No □ Date that this symptom started (DD/MM/YYYY): __/__/____ Date that this symptom ended (leave in blank if symptom is ongoing) (DD/MM/YYYY): __/__/____ Duration of symptom (days):
Did any of these symptoms require you to seek medical attention?	Yes □ No □
Did any of these symptoms require you to miss work or school?	Yes □ No □
Did any of these symptoms require you to be hospitalized	Yes □ No □
Loss of sense of smell	Yes □ Severity (Low/Medium/High) No □ Date that this symptom started (DD/MM/YYYY): __/__/____ Date that this symptom ended (leave in blank if symptom is ongoing) (DD/MM/YYYY): __/__/____ Duration of symptom (days):
Loss of sense of taste	Yes □ Severity (Low/Medium/High) No □ Date that this symptom started (DD/MM/YYYY): __/__/____ Date that this symptom ended (leave in blank if symptom is ongoing) (DD/MM/YYYY): __/__/____ Duration of symptom (days):
Severity of symptoms 1–10:	
Other symptoms/ Comments:	

**Table A2:** Food Frequency Questionnaire FFQ.

Food	How Many Times?	
	in a Week	in a Month
Milk		
Yogurt		
Chocolate: “Kit Kat”, “Mars”…		
Breakfast cereales (“Corn-Flakes”, “Kellog’s”)		
Biscuits		
Biscuits with chocolate or cream		
Cupcakes, spongecakes		
Donut, crossiants		
Salads: Lettuce, tomato…		
Green beans, chard, spinach		
Garnish vegetables: eggplant, mushrooms		
Baked, fried or boiled potatoes		
Legumes: lentils, chichpeas, beans		
White rice, paella		
Pasta: noodles, macarrones, spaguetti		
Soups or creams of soups		
eggs		
Chichen or turkey		
beef, pork, lamb (steak, pie,…)		
Mince meat, sausage, hamburger		
White fish: Hake, grouper,…		
Blue fish: sardines, tuna, salmon,…		
Shellfish: muscles, prawns, squid,…		
Croquettes, dumpings, pizza		
bread (in baguette, with meals,…)		
Salted/cured ham, sliced ham, sausages		
White or fresh cheese (Philadelphia) o low in calories		
Other cheese: cured or semicured, creamy		
Fruititricic: orange, mandarine,…		
Other fruits: apple, pear, peach, banana…		
Preserved fruits (in syrup…)		
Natural fruit juices		
Concentrated fruit juices		
Nuts: peanuts, hazelnuts, almonds,…		
Milky desterts: custard, flan, cottage cheese		
Cream or chocolate cakes		
Packets of crisps		
sweets: gummies, candies…		
Ice cream		
Fizzy sμgar drinks (“coca-cola”, “Fanta”…)		
Low calorie fizzy drinks (coca-cola light…)		
Wine, sangria		
Beer		
Beer 0% alcohol		
Distilled drinks: whisky, gin, coñac,…		

**Table A3:** FFQ analysis comparison across the 3 symptom groups.

Food Group	Asymptomatic	Symptomatic	Long-Term COVID
Eggs	2.0 (0.0–14.0), 2.7	2.5 (0.0–6.0), 2.3	2.0 (0.0–14.0), 1.9
Whitefish: Hake, grouper…	1.0 (0.0–2.0), 0.8	1.0 (0.0–3.5), 1.6	1.0 (0.0–3.0), 0.9
cheese: cured or semi cured, creamy	2.0 (0.8–7.0), 3.0	1.0 (0.0–12.0), 3.0	1.0 (0.0–18.5), 2.2
Citric fruits: mandarin, orange...	1.0 (0.0–14.0), 1.6	1.0 (0.0–14.0), 5.4	2.0 (0.0–14.0) 6.4
Other fruit: apple, pear, peach banana	5.5 (0.0–28.0), 5.4	4.0 (0.5–18.0), 6.0	5.5 (0.0–18.0), 5.9
Natural fruit juices	0.3 (0.0–7.0), 3.3	0.0 (0.0–2.0), 0.6	0.1 (0.0–7.0), 1.0
Fizzy sμgared drinks	0.2 (0.0–4.0), 1.0	0.0 (0.0–3.8), 0.6	0.0 (0.0–5.0), 0.0
Beer	2.4 (0.0–7.0), 3.6	1.0 (0.0–8.8), 5.1	1.0 (0.0–8.8), 2.8
Yogurt	1.1 (0.0–4.0), 2.6	1.0 (0.0–7.0), 3.0	2.0 (0.0–8.0), 2.4
Milk	5.3 (0.0–14.0), 6.4	0.3 (0.0–7.0), 7.0	7.0 (0.0–14.0), 6.8
Chocolate: “Kit Kat”, “Mars” …	1.0 (0.0–10.0), 1.2	1.0 (0.0–3.0), 1.8	0.4 (0.0–2.0), 0.8
Breakfast cereals: “Corn-Flakes”, “Kellogg’s”	0.0 (0.0–5.0), 1.1	0.0 (0.0–1.4), 0.3	0.0 (0.0–2.0), 0.0
Doμghnuts, Croissants	0.3 (0.0–2.5), 0.5	0.0 (0.0–2.0), 0.5	0.0 (0.0–3.0), 0.3
Salads: Lettuce, tomato…	5.0 (0.0–7.0), 5.0	4.5 (0.0–7.0), 4.5	4.0 (0.0–14.0), 4.8
Green beans, chard, spinach	1.0 (0.0–7.0), 1.6	1.0 (0.0–7.0), 2.3	1.0 (0.0–7.0), 1.7
Baked, fried, or boiled potatoes	1.6 (0.0–4.0), 1.2	1.3 (0.0–6.0), 2.0	2.0 (0.0–7.0), 2.0
Legumes: lentils, chickpeas, beans	1.0 (0.3–2.0), 0.7	2.0 (0.3–6.5), 2.2	1.8 (0.0–9.3), 1.4
Pasta: noodles, macaroni, spaghetti	1.0 (0.5–3.5), 1.2	1.3 (0.3–5.0), 1.7	1.0 (0.3–7.0), 0.0
Beef, pork, lamb (steak, pie...)	1.0 (0.0–5.0), 1.9	1.0 (0.0–2.0), 1.9	1.0 (0.0–3.0), 1.8
Mincemeat, sausage, hamburger	1.0 (0.0–5.0), 1.8	1.0 (0.0–3.0), 1.6	1.0 (0.0–14.0), 1.5
Bluefish: sardines, tuna, salmon...	0.6 (0.0–2.0), 1.0	1.0 (0.0–7.0), 0.5	1.0 (0.0–7.0), 0.8
Shellfish: muscles, prawn, squid...	0.5 (0.0–1.0), 0.9	0.5 (0.0–1.3), 1.0	0.5 (0.0–4.0), 1.0
Bread (in baguette, with meals...)	6.0 (0.0–22.0), 5.3	4.0, (0.0–7.0), 4.5	7.0 (0.0–14.0), 3.9
Salted/cured ham, sliced ham, sausages	2.5 (0.0–12.0), 4.1	2.0 (0.0–7.0), 3.9	2.5 (0.0–21.0), 5.3
Nuts: peanuts, hazelnuts, almonds…	2.0 (0.0–10.0), 4.3	3.0 (0.0–10.5), 3.8	1.0 (0.0–12.3), 2.5
Distilled drinks: Whiskey, gin, cognac…	0.0 (0.0–1.1), 0.6	0.3 (0.0–2.3), 0.9	0.0 (0.0–3.0), 0.7

Data are reported as median (range), IQR portions per week. No significance was observed between the consumption of any of these food groups.

**Table A4:** (a) Median and IQR antibody concentrations for different age groups. (b) Median and IQR antibody concentrations for each symptom group.

(**a**)	
**Age Group**	**Median (Minimum–Maximum), IQR IgG Concentration BAU/mL**
≤25 (n = 18)	109 (35–296), 135 ^a^
26–35 (n = 17)	58 (0–334), 137 ^b^
≥36 (n = 20)	42 (0–502), 69 ^b^
(**b**)	
**Symptom Group**	**Median (Minimum–Maximum), IQR IgG Concentration BAU/mL**
Asymptomatic (n = 20)	35 (0–296), 86 ^a^
Symptomatic (n = 14)	78 (0–239), 163 ^ab^
Long-term COVID (n = 21)	108 (0–502), 130 ^b^

(a) A comparison of antibody response among different age groups. Those not sharing same superscript within the column were significantly different at *p* ≤ 0.05 in the Mann–Whitney U test. (b) A comparison of antibody response among the different symptom groups. Those not sharing same superscript within the column were significantly different at *p* ≤ 0.05 in the Mann–Whitney U test.

**Table A5:** Characteristics and food diary analysis of the 12 participants for whom antibody results are available on two different occasions over the 6-month period. Exclamation marks show significant deficiencies below the RDI.

Participant	Gender	Age	BMI	Supplements	Symptom Group	Fe (RDI)	Selenium (RDI)	Zinc (RDI)	Vitamin A (RDI)	Vitamin D (RDI)	Vitamin E (RDI)	Vitamin B2 (RDI)	Vitamin B6 (RDI)	Vitamin B9 (RDI)	Vitamin B12 (RDI)	Vitamin C (RDI)	Carotene	FFQ Citric Fruits *p*/*w*	FFQ Other Fruits *p*/*w*
N	F	29	22.3	C5 Alfa plus	S	7.0 mg (47%)!	42 μg (69%)	5.9 mg (73%)	515 μg (42%)	14.1 μg (93%)	11.8 mg (84%)	1.2 mg (105%)	1.2 mg (100%)	170 μg (37%)!	5.5 μg (229%)	47 mg (24%)	1490 μg (29%)	0.3	3.0
T	F	43	22.3	*C.sinensis*	S	8.8 mg (59%)	85 μg (140%)	5.9 mg (73%)	419 μg (34%)	3.0 μg (19%)!	7.1 mg (50%)	0.8 mg (75%)	1.3 mg (110%)	133 μg (29%)!	3.1 μg (130%)	36 mg (19%)!	1049 μg (21%)	7.0	7.0
B	F	23	22.3	No	LC	5.8 mg (39%)!	37.4 μg (62%)	5 mg (62%)	258 μg (21%)	0.72 μg (4%)!	3.6 mg (25%)	0.5 mg (48%)!	1 mg (84%)	130 μg (28%)!	2 μg (85%)	92 mg (48%)	1267 μg (25%)	7.0	14.0
D	F	26	20.2	No	AS	12.7 mg (79%)	45 μg (64%)	4.4 mg (40%)	617 μg (95%)	12.6 μg (84%)	10.9 mg (99%)	0.6 mg (38%)	2.3 mg (142%)	196 μg (59%)	4 μg (99%)	143 mg (150%)	2462 μg (N/A)	1.0	0.0
E	F	29	30.8	No	AS	17.5 mg (118%)	89 μg (148%)	11.9 mg (148%)	480 μg (39%)	14.6 μg (97%)	14.1 mg (101%)	2.6 mg (233%)	2.9 mg (240%)	220 μg (49%)!	9.7 μg (406%)	54 mg (28%)	2062 μg (41%)	0.5	0.8
F	F	41	25.8	EPPlus	AS	8.2 mg (55%)	52 μg (87%)	5.8 mg (73%)	656 μg (53%)	2.2 μg (14%)!	8.2 mg (58%)	2.1 mg (186%)	2.2 mg (179%)	239 μg (53%)!	5.8 μg (240%)	50 mg (26%)	874 μg (17%)	0.0	5.0
H	F	35	23.1	No	AS	4.5 mg (30%)!	57 μg (94%)	4 mg (49%)	260 μg (21%)	1.5 μg (10%)!	3.7 mg (26%)	0.9 mg (79%)	0.8 mg (65%)!	126 μg (28%)!	4.4 μg (184%)	51 mg (27%)	626 μg (12%)	2.0	7.0
K	F	23	21.5	No	AS	11.1 mg (75%)	67 μg (110%)	7.5 mg (94%)	746 μg (61%)	2.8 μg (18%)!	8.9 mg (63%)	1.4 mg (123%)	1.6 mg (135%)	186 μg (41%)!	4.3 μg (180%)	34.8 mg (18%)!	2412 μg (48%)	2.0	14.0
O	M	24	24.8	No	LC	7.9 mg (90%)	77 μg (102%)	10.2 mg (107%)	237 μg (33%)!	2.5 μg (16%)!	5 mg (63%)	1.1 mg (85%)	2.0 mg (141%)	157 μg (78%)	5.8 μg (383%)	69 mg (171%)	838 μg (N/A)	0.5	0.5
U	M	30	24.6	No	LC	16.1 mg (184%)	36.9 μg (52%)!	10.7 mg (112%)	937 μg (133%)	15.2 μg (101%)	18.7 mg (230%)	1.6 mg (122%)	2.8 mg (199%)	296 μg (147%)	5.7 μg (380%)	103 mg (256%)	1068 μg (N/A)	1.0	7.0
V	M	25	24.9	MVC	S	12.8 mg (146%)	24.8 μg (33%)!	5.5 mg (57%)!	329 μg (46%)	7.0 μg (46%)!	5.3 mg (65%)	0.8 mg (61%)!	0.8 mg (55%)!	235 μg (117%)	1.0 μg (66%)	126 mg (315%)	2079 μg (N/A)	3.0	18.0
Z	F	25	22	No	S	5.2 mg (35%)!	59 μg (99%)	5.7 mg (71%)	454 μg (37%)	0.3 μg (2%)!	5.4 mg (38%)	1 mg (94%)	0.8 mg (69%)	149 μg (33%)!	4.8 μg (200%)	31 mg (16%)!	1800 μg (36%)	0.25	7.0

Note, individuals with increasing Ab concentration (N and T) have been placed at the top of the table. FFQ is reported in portions per week. MVC—Multivitamin complex, EPPlus—ErgyPhilus.
